# 

**Published:** 1892-01-16

**Authors:** 


PRICE ONE PENNY.

No. 277, Vol. XI.—January 16th, 1892.

[Registered at the General Post Office as a Newspaper for transmission in the United Kingdom and Abroad.]

WITH EXTRA NURSING SUPPLEMENT.
Per Annum, 6s. 6d., post free.
Monthly Parts, 6d.; post free, 8d.
Ditto per Annum, 8s., post free.

### THE HOSPITAL

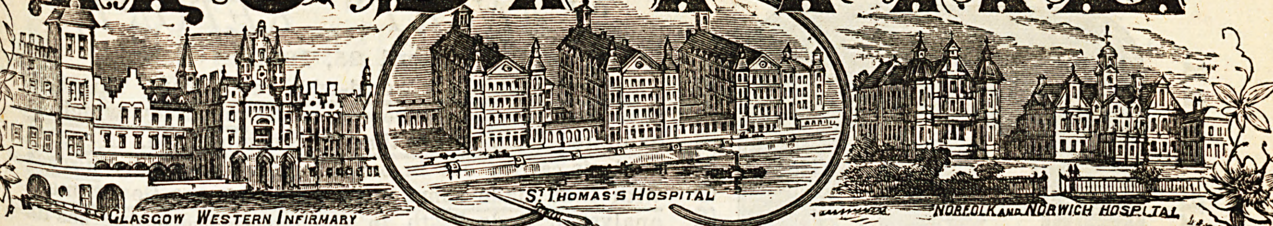

A WEEKLY JOURNAL OF

### Science, Medicine, Nursing, and Philanthropy.

SATURDAY, JANUARY 16th, 1892.

**Influenza Specifics** . . . 189
**Passing Topics.**—The Harvest of the Sick Poor . . . 190
**The Doctor's Armchair.**—Writers' Brains . . . 191
**Some Scientific Aspects of Insanity.** IX.—Mania . . . 192
**Some American Hospitals.** III.—Salt Lake City . . . 193
**Everybody's Page** . . . 193
**Notes and News** . . . 194
**General Charities** . . . 195
**Annotations.**—Lady Smokers—Influenza Patients in General Hospitals—Oxford Buckles on its Armour . . . 196
**Turning over New Leaves.**—Three Weeks in a Hospital—Electricity up to Date . . . 200
**The Practitioner's Mirror and Retrospect**—
MEDICINE.—Diseases of the Arteries, Veins, and Capillaries—On Some Urinary Tests I. . . . 197
SURGERY.—Barker's Method of Excising the Hip Joint in Tuberculous Disease . . . 198
PRACTITIONER'S BOOKSHELF.—First Lines in Midwifery—Savill's Prescribers' Companion—Letts's Medical Diary . . . 199
NEW DRUGS, APPLIANCES, AND THINGS MEDICAL.—New Clinical Thermometer: Hicks' Patent . . . 200
**Scraps and Gleanings** . . . 195
**The Student of Medicine.**—Appointments—Vacancies—Notes from the Schools—Pass Lists.

EXTRA SUPPLEMENT.—THE NURSING MIRROR.

**En Passant.**—Derby Nursing and Sanitary Association—Presentations—A Christmas Pudding—Village Nursing—Red Cross Nurses—Short Items—Infirmary Nursing . . . xci

**The Expression of the Eye** . . . xcii

**Everybody's Opinion.**—Christmas Fare in Hospitals—Concerts in Hospitals—Queen's Nurses . . . xcii

**Real Scandals** . . . xciii

**Appointments** . . . xciii

**For Reading to the Sick.**—Anniversaries . . . xciii

**A Vegetarian Dinner** . . . xciv

**Keeping Christmas** . . . xciv

**Presentations** . . . xcv

**Notes and Queries** . . . xcv

**Off Duty.**—Told on New Year's Eve (*concluded*) . . . xcvi

